# Integrative DNA methylome and transcriptome analysis identify potential genes on the influence of dilated cardiomyopathy-associated heart failure

**DOI:** 10.1186/s13148-025-01876-2

**Published:** 2025-04-28

**Authors:** Zhenglong Guo, Yunfei Liu, Zhiming Zhou, Jianchao Chen, Lin Guo, Keke Liang, Yibin Hao, Bingtao Hao, Bin Yang, Shixiu Liao

**Affiliations:** 1 Henan Provincial Key Laboratory of Genetic Diseases and Functional Genomics and Medical, Genetics Institute of Henan Province, Henan Provincial People’s Hospital, People’s Hospital of Zhengzhou University, Zhengzhou, China; 2 School of Medicine, People’s Hospital of Henan University, Henan University, Zhengzhou, China; 3 Henan Key Laboratory of Cardiac Remodeling and Transplantation, Zhengzhou Seventh People’s Hospital, Zhengzhou, China; 4 Department of Critical Care Medicine, Zhengzhou Seventh People’s Hospital, Zhengzhou, China; 5 Department of Immunology, School of Basic Medical, Zhengzhou University, Zhengzhou, China; 6 Eye Institute, Henan Academy of Innovations in Medical Science, Zhengzhou, Henan China

**Keywords:** DNA methylation, RNA-seq, WGBS, Heart failure, Atrial tissue, Dilated cardiomyopathy

## Abstract

**Objective:**

Dilated cardiomyopathy (DCM)-associated heart failure (HF) presents a significant clinical challenge, underlying epigenetic mechanisms remaining poorly understood. This study aims to investigate the interplay between DNA methylation and gene expression in the hearts of patients with DCM-associated HF (DCM-HF).

**Methods:**

Atrial tissues were collected from five healthy donors and five heart transplant recipients suffering from heart failure due to DCM. We conducted RNA-sequencing (RNA-seq) to analyze mRNA expression profiles and performed whole-genome bisulfite sequencing (WGBS) to evaluate DNA methylation levels. Correlation analyses between RNA-seq and WGBS data were executed by integrating differentially expressed genes (DEGs) with genes associated with differentially methylated regions (DMRs) located in the promoter regions.

**Results:**

The RNA-seq analysis identified a total of 681 DEGs, comprising 406 significantly downregulated genes and 275 upregulated genes in DCM-HF tissues, which were enriched in pathways related to cardiomyopathy. WGBS revealed 16,158 hypomethylated and 6,857 hypermethylated differentially methylated regions (DMRs), with 3,185 of these located in promoter regions. The integration of promoter-hypomethylated and hypermethylated DMRs-related genes (DMGs) with DEGs resulted in the identification of 46 hub genes associated with cardiac development and function. Protein–protein interaction and disease association analyses highlighted five key genes—*NPPA*, *NPPB*, *ACTN2*, *NEBL*, and *MYO18B*-that exhibited promoter hypomethylation and increased expression, potentially linked to the activity of transcription factors such as HIF1A and KLF4.

**Conclusions:**

These findings suggest that the epigenetic dysregulation of cardiac stress-response and structural genes contributes to the pathogenesis of DCM-HF. Furthermore, the detection of promoter methylation levels in these loci may offer new opportunities for developing diagnostic tools and therapeutic strategies for DCM-HF management.

**Supplementary Information:**

The online version contains supplementary material available at 10.1186/s13148-025-01876-2.

## Introduction

Dilated cardiomyopathy (DCM) is a common cause of heart failure characterized by left ventricular dilation and impaired systolic function, which leads to significant morbidity and mortality [1–3]. Despite advances in medical and surgical treatments, the underlying molecular mechanisms driving DCM-associated heart failure (DCM-HF) remain inadequately understood. DNA methylation, an important epigenetic modification, involves the addition of a methyl (CH3) group to the cytosine base of DNA, primarily in cytosine-guanine dinucleotides (CpGs) [4]. This modification dynamically regulates gene expression, and its potentially reversible nature in response to biological and environmental factors makes it a valuable indicator for predicting disease risk and prognosis of outcomes, including cancer, obesity, and aging [5–7].

Emerging evidence has linked changes in DNA methylation to the risk and outcomes of cardiovascular diseases (CVDs), including congenital heart defects [8], hypertension [9], myocardial infarction (MI) [10], stroke [11], and dilated cardiomyopathy (DCM) [12]. These associations may be attributed to the modulation of gene expression related to cardiac function, metabolism, and inflammation. For instance, differentially methylated regions (DMRs) at the *ZBTB12* gene and *LINE-1* elements in white blood cells have been associated with an increased risk of myocardial infarction (MI) [13]. Furthermore, aberrant DNA methylation has been shown to significantly alter the mRNA expression of *LY75* and *ADORA2A*, which contribute to the development and progression of DCM [14].

Many studies have demonstrated that DNA methylation patterns can significantly influence the severity and prognosis of heart failure; however, the specific regulatory mechanisms involved remain inadequately understood [15–17]. It is widely acknowledged that DNA methylation in promoter regions modulates gene expression through various mechanisms, including the inhibition of transcription factor binding, alterations to chromatin structure, synergistic interactions with histone modifications, and effects on transcription initiation [18]. In this study, we performed RNA-seq and whole-genome bisulfite sequencing (WGBS) on atrial tissues obtained from healthy donors and heart transplant recipients to identify differentially expressed genes regulated by promoter methylation. These analyses aim to uncover potential biomarkers and therapeutic targets for dilated cardiomyopathy-associated heart failure (DCM-HF).

## Methods

### Human sample collection

Atrial tissue samples were collected from both recipients and donors at the Heart Transplant Center of Zhengzhou Seventh People’s Hospital (Zhengzhou Cardiovascular Hospital). This study received approval from the Medical Ethics Committee of Zhengzhou Seventh People’s Hospital (Approval No: 2020-012). Written informed consent detailing the research objectives involving human material was obtained from all patients or their families. The donors were male individuals who had experienced brain death due to cerebral hemorrhage or traffic accidents. The recipient hearts were sourced from males diagnosed with end-stage heart failure resulting from dilated cardiomyopathy (DCM), in accordance with the guidelines established by the European Society of Cardiology (ESC) [19, 20].

### RNA-sequencing

Total RNA was extracted from atrial tissues using Trizol reagent (Invitrogen, USA). The integrity and concentration of the extracted RNA were assessed with an Agilent 2100 Bioanalyzer (Agilent Technologies, CA, USA). High-quality complementary DNA (cDNA) libraries were constructed and sequenced on Novaseq X (Illumina) to generate raw sequencing reads. The raw data were filtered using Fastp to remove adaptor sequences, unknown reads containing poly-N sequences, and low-quality reads, resulting in a set of clean reads. These clean reads were subsequently aligned to the human reference genome (hg38) using HISAT2 v2.0.5. Differential expression analysis was performed with DESeq2, applying thresholds of a false discovery rate (FDR) of a false discovery rate (FDR) < 0.05 and |log2 (fold change)|> 1 to identify significantly differentially expressed genes (DEGs). An online platform (https://www.bioinformatics.com.cn) was utilized for data analysis and visualization, encompassing Gene Ontology (GO), Kyoto Encyclopedia of Genes and Genomes (KEGG), and Gene Set Enrichment Analysis (GSEA).

### Whole-genome bisulfite sequencing

DNA was extracted from tissue samples using the FastPure Tissue DNA Isolation Mini Kit (#DC112, Vazyme, China) following the manufacturer’s instruction. 100 ng of genomic DNA was combined with 0.5 ng of unmethylated lambda DNA and subsequently fragmented to a size range of 200–400 bp using an ultrasonic crusher (Covaris S220, USA). Following fragmentation, unmethylated cytosines were converted to uracils using the EZ DNA Methylation-Gold™ kit (Zymo Research). The constructed library, with an effective concentration exceeding 1.5 nM, was assessed using a Bioanalyzer (Agilent 5400, USA), quantified via Q-PCR, and sequenced on the Novaseq X platform (Illumina). The quality of the raw data was evaluated using FastQC (v0.11.8), and the data were further filtered to obtain clean data through fastp software (v0.23.1). This clean data was subsequently aligned to the reference human genome (hg38) using Bismark software (v0.24.0). Binary tests were performed to identify methylation sites by analyzing the number of methylated cytosines (mC), the total number of cytosines (the sum of mC and unmethylated cytosines, umC), and the non-conversion rate (r). A site was classified as methylated when the false discovery rate (FDR) was less than 0.05. Differentially methylated regions (DMRs) were subsequently analyzed using DSS software (v2.12.0).

### Protein–protein interaction analysis

The protein–protein interaction network was constructed utilizing the STRING database (version 12.0, https://string-db.org). A gene list was uploaded to the STRING platform, with Homo sapiens designated as the target organism. Interactions were predicted based on a combination of curated databases, experimental evidence, co-expression data, and text mining approaches. Functional enrichment analysis of hub genes was conducted using the STRING database, filtering for significance in Gene Ontology (GO) terms and Disease-gene Associations (DISEASES) with a false discovery rate (FDR) threshold of less than 0.05.

### Quantitative real-time PCR (RT-qPCR)

Total RNA was extracted from atrial tissues of various groups using TRIzol (Invitrogen, USA) in accordance with the manufacturer’s instructions. The quality and concentration of the RNA were assessed using a Nanodrop spectrophotometer (Thermo Fisher Scientific, USA), and cDNA was synthesized using the RevertAid RT kit (Thermo Fisher Scientific, USA). Real-time PCR was conducted on the Applied Biosystems StepOnePlus qPCR system utilizing SYBR qPCR Master Mix (#Q712, Vazyme, China). The relative mRNA expression was quantified using the 2 − ΔΔCt method, normalizing to actin-beta (ACTB), with the primers shown in Table S1.

### Statistical analysis

Data were presented as the means ± SEM, and two-tailed Student’s t-test was used to analyze statistical differences between two groups. *P* < 0.05 was considered statistically significant.

## Results

### Clinical characteristics of recipients and donors

**Fig. 1:**
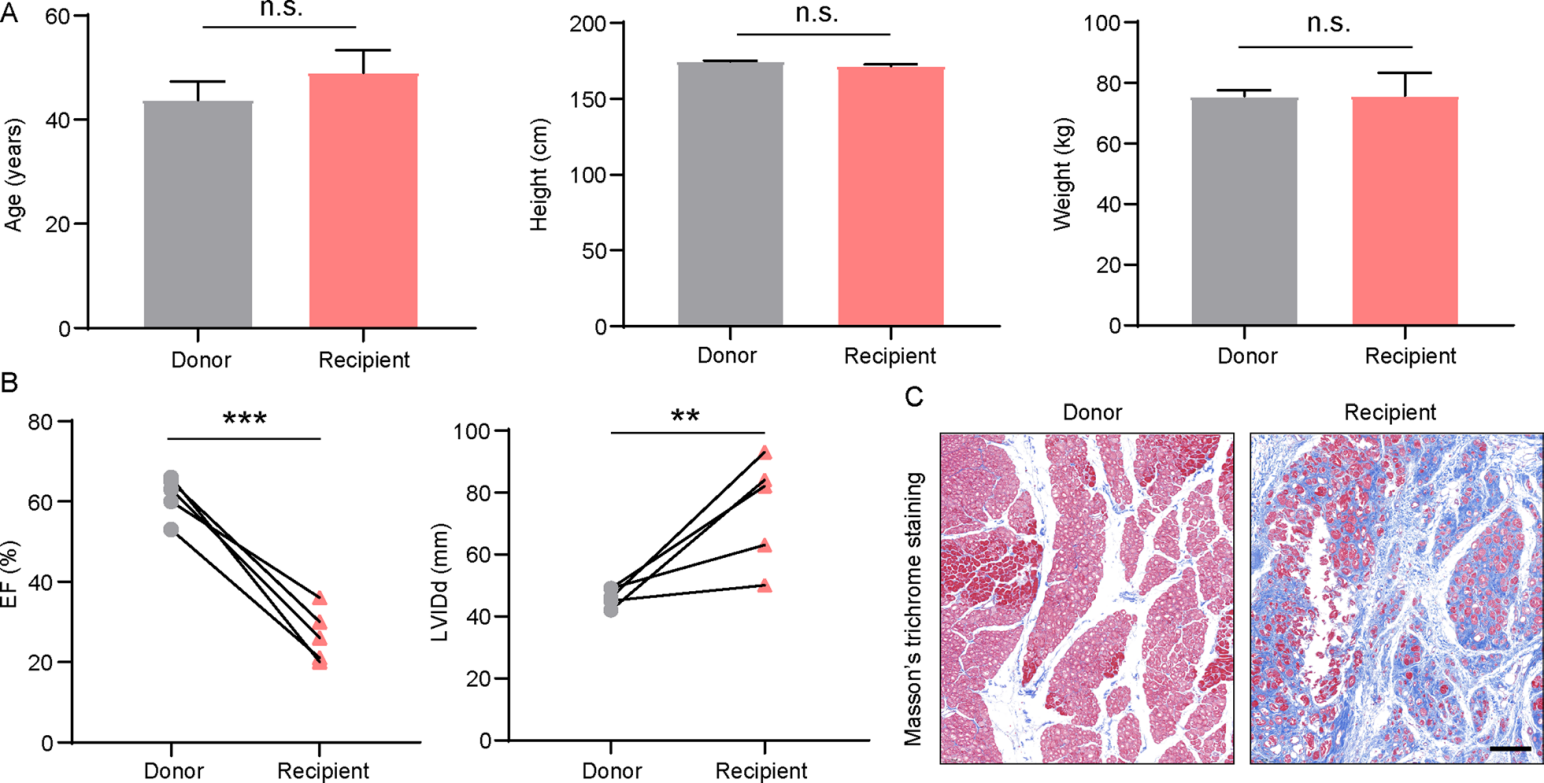
Cardiac function and pathological manifestations in DCM-HF. **A** Basic clinical information of the patients. **B** Echocardiographic functional assessment of hearts from healthy donors and heart transplant recipients with dilated cardiomyopathy-associated heart failure (DCM), including ejection fraction (EF) and left ventricular end-diastolic dimension (LVIDd). Data are presented as means ± SEM (n = 5 samples per group), **P < 0.01; ***P < 0.001. **C** Masson’s trichrome staining of atrial tissues from donor and recipient hearts. Scale bar = 100 μm

**Fig. 2:**
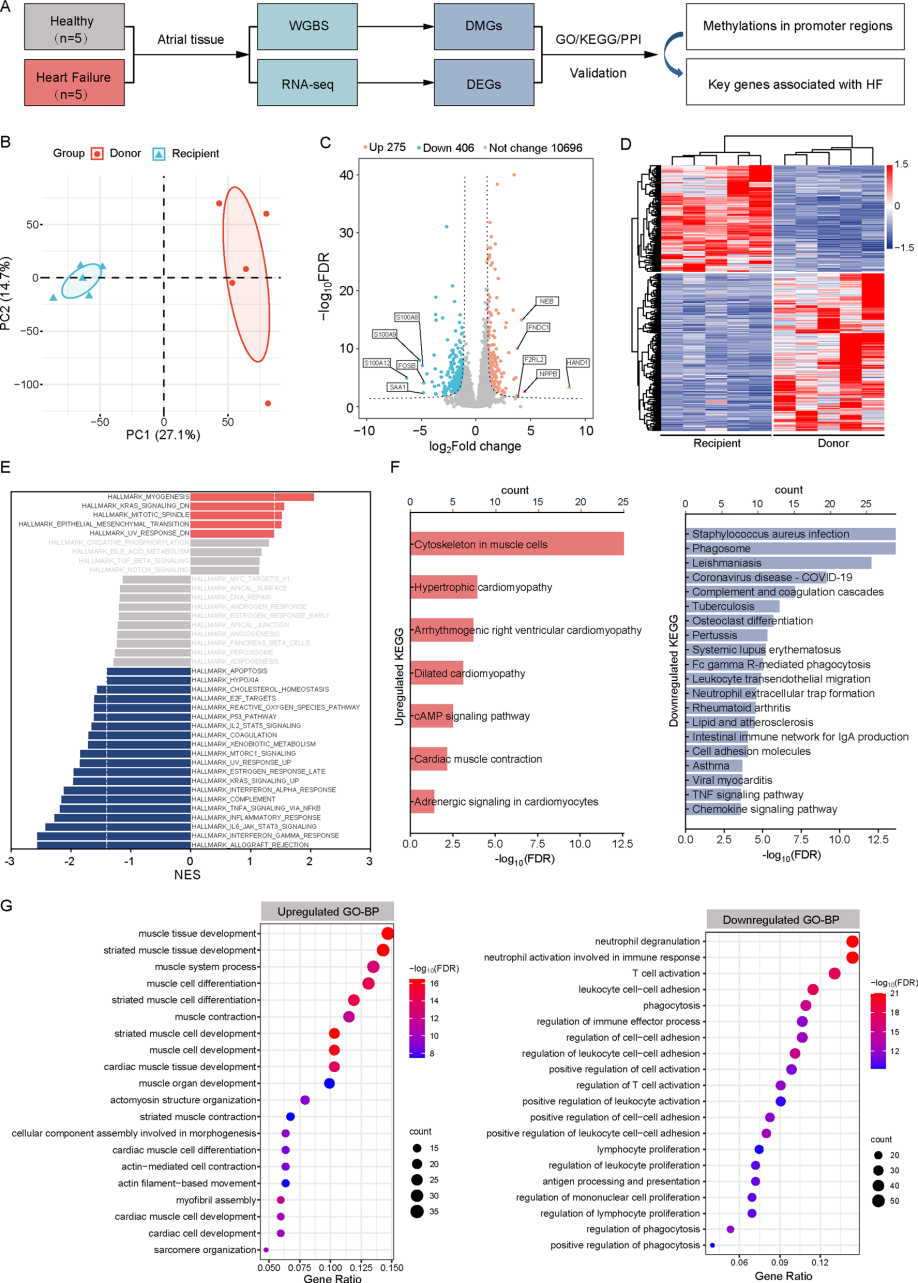
RNA-sequencing analysis of atrial tissues from the Donor and Recipient groups. **A** General workflow used in this study. **B** Principal Component Analysis (PCA) of mRNA expression profiles in the hearts of donors and recipients. **C** Volcano plots depicting the distribution of differentially expressed genes (DEGs). **D** Heatmaps of 681 DEGs with |log2FC|> 1 and FDR < 0.05. **(E)** Gene Set Enrichment Analysis (GSEA) of expressed genes in Recipient versus Donor. **F-G** Kyoto Encyclopedia of Genes and Genomes (KEGG) (F, upper) and Gene Ontology (GO)-Biological Process (BP) enrichment (G, lower) of the significantly up- and downregulated DEGs

Recipients of heart transplant surgery due to end-stage heart failure, primarily resulting from dilated cardiomyopathy (DCM), were identified based on their medical history, cardiac ultrasound, and serum biomarkers including N-terminal pro-B-type natriuretic peptide (NT-proBNP). Analysis of clinical data revealed no significant differences in age, height, and weight between the recipients and healthy donors (Fig. 1A). However, echocardiographic assessments demonstrated that the left ventricular ejection fraction (LVEF) in recipients was significantly lower than that observed in donors, with all recorded values falling below 40%. Additionally, left ventricular end-diastolic internal dimensions (LVIDd) were significantly increased in recipients (Fig. 1B). Further analysis using Masson’s trichrome staining on atrial tissues revealed pronounced myocardial fibrosis in the hearts of recipients (Fig. 1C). Collectively, these findings suggest that patients with DCM-HF exhibit significant pathological alterations and cardiac dysfunction. In preparation for transcriptomic and DNA methylation analyses, we obtained direct samples of left atrial tissue without appendage, which are expected to provide a more accurate representation of the overall state of the entire atrium. In this study, samples from five recipient hearts and five donor hearts were collected, and the detailed research process is shown in Fig. 2A.

### Gene expression profile of DCM-HF by RNA-seq

**Fig. 3:**
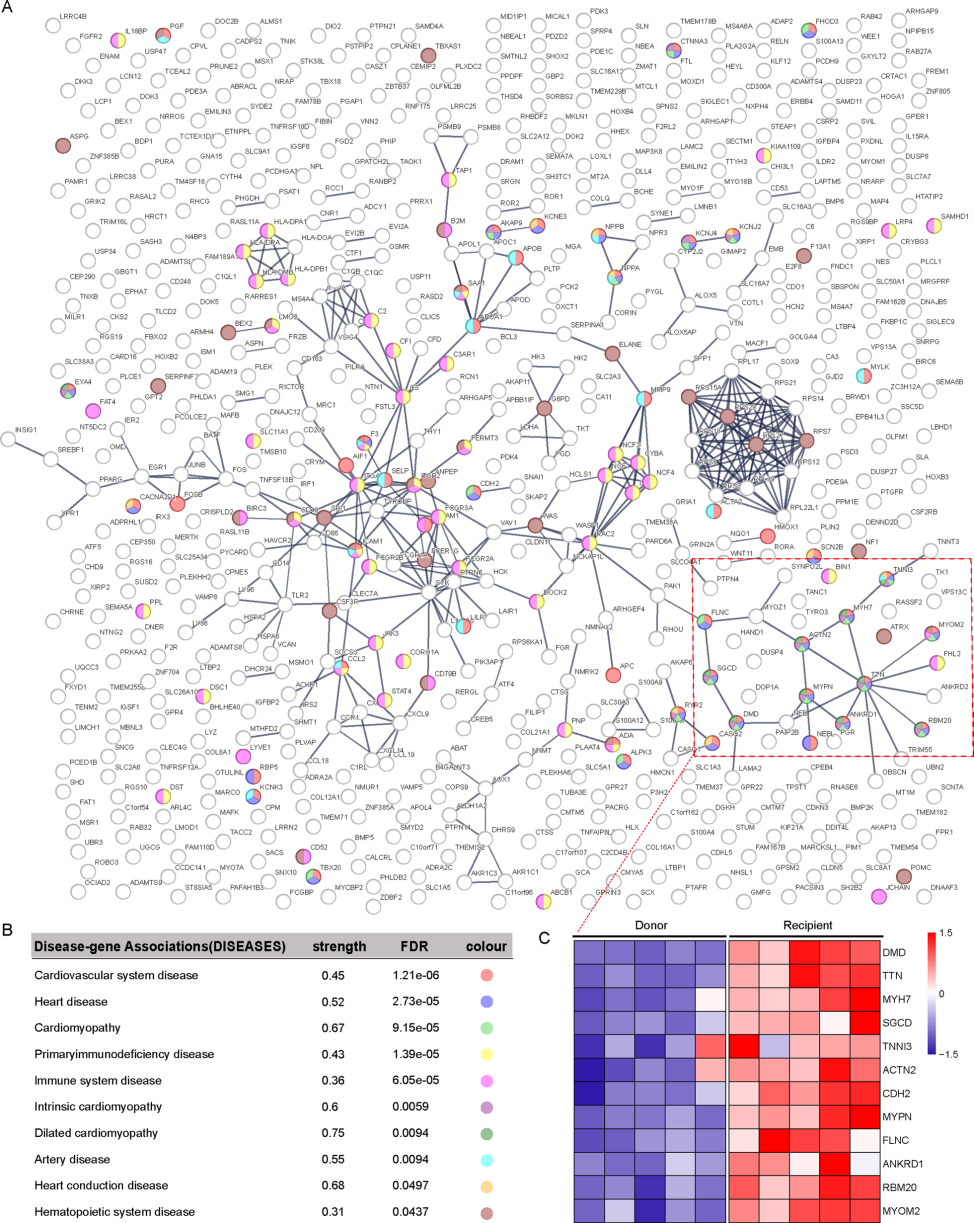
Screening of candidate differentially expressed genes (DEGs) implicated in the pathogenesis of heart failure. **A** and **B** The Protein–protein Interaction (PPI) network (A, upper) and the Disease-gene Associations (DISEASES) prediction (B, lower) among the DEGs constructed using the STRING online tool. **C** Heatmaps of 12 DEGs associated with heart diseases

Principal Component Analysis (PCA) and differential gene expression analysis reveal significant difference in expression patterns between recipients and donors, identifying a total of 681 differentially expressed genes (DEGs) (Fig. 2B and C, Table S2). Among these, 275 genes are significantly upregulated while 406 genes are significantly downregulated (Fig. 2D) in the recipients. To further elucidate the functional implications of these DEGs and their impact on the heart, we conducted enrichment analyses utilizing Gene Set Enrichment Analysis (GSEA), Gene Ontology-Biological Process (GO-BP), and Kyoto Encyclopedia of Genes and Genomes (KEGG) methodologies. The results indicate that the upregulated DEGs are predominantly associated with cardiac development, differentiation, structural integrity, and contractile function, and are implicated in conditions such as hypertrophic cardiomyopathy and dilated cardiomyopathy. In contrast, the downregulated DEGs are linked to the proliferation, activation, and interaction of immune cells, with associations to inflammatory pathways including TNF-α signaling and immune responses (Fig. 2E–G). To identify key genes among the candidate DEGs, we employed the String online tool to construct an interaction network (Fig. 3A). Through Disease-gene Association (DISEASES) analysis, we identified 12 pivotal genes associated with various cardiovascular diseases, including dilated cardiomyopathy, heart conduction disease, and artery diseases. These key genes include *DMD*, *TTN*, *MYH7*, *SGCD*, *TNNI3*, *ACTN2*, *CDH2*, *MYPN*, *FLNC*, *ANKRD1*, *RBM20*, and *MYOM2*. Notably, the expression of these genes is markedly elevated in recipients (Fig. 3B and C). These findings indicate that the expression patterns in recipient hearts have been altered, suggesting that the upregulated DEGs may contribute to the remodeling and dysfunction of cardiomyocytes associated with DCM-HF.

### DNA methylation landscape of DCM-HF by WGBS

**Fig. 4:**
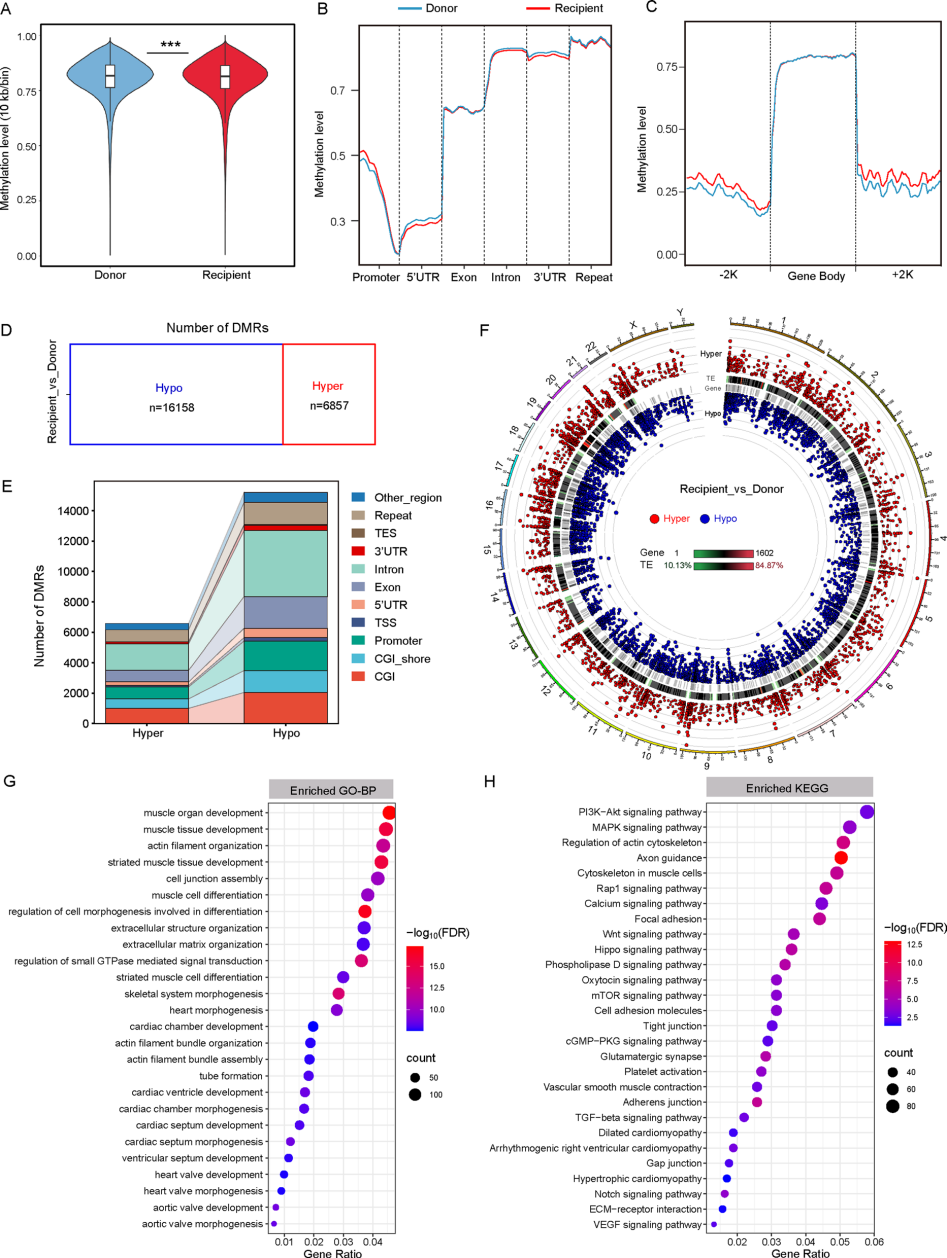
Whole-Genome Bisulfite Sequencing (WGBS) analysis of atrial tissues from the Donor and Recipient groups. **A** Violin plots showing the global DNA methylation levels of CpG sites in recipient hearts compared to donors. (n = 5 samples per group, ***P < 0.001).** B** Methylation levels across various genomic functional regions in Recipient versus Donor. **C** Methylation patterns of the gene body, 2 kb upstream of the transcription start site (TSS) and 2 kb downstream of the transcription end site (TES) in each group. **D** The ratio of hypermethylated and hypomethylated Differentially Methylated Regions (DMRs) in Recipient versus Donor. **E** Proportions of DMRs located in different genomic features. **F** The distribution of DMRs across the whole-genome. **G** and **H** GO-BP enrichment (G, left) and KEGG analysis (H, right) of DMR-related Genes (DMGs)

Whole-genome bisulfite sequencing (WGBS) was employed to construct a comprehensive genome-wide DNA methylation landscape [21]. The results indicate that the average DNA methylation level in the recipient group is lower than that observed in the donor group (Fig. 4A). A comparative analysis of methylation levels across various genomic functional regions revealed an increase in DNA methylation within the promoter region of the recipient group, while methylation levels in the 5’ untranslated region (UTR) and 3’ UTR showed a decrease (Fig. 4B). Furthermore, while the methylation levels within the gene body remained largely unchanged, those located 2 kb upstream of the transcription start site (TSS) and 2 kb downstream of the transcription termination site (TES) exhibited an increase (Fig. 4C). In total, 23,015 differentially methylated regions (DMRs) were identified, comprising 16,158 hypomethylated DMRs and 6,857 hypermethylated DMRs (Fig. 4D). These DMRs are distributed across various genomic functional regions, including promoter regions, exon regions, intron regions, CpG islands (CGIs), CGI shores, and repeat regions (Fig. 4E). Notably, these DMRs encompass 3,630 genes and are distributed across all chromosomes (Fig. 4F). Consistent with transcriptome results, GO-BP and KEGG enrichment analyses revealed that the differentially methylated region-related genes (DMGs) predominantly influence biological processes associated with cardiac development and function (Fig. 4G). Additionally, these genes are implicated in conditions such as hypertrophic cardiomyopathy, dilated cardiomyopathy, and cell adhesion, as well as in pathways related to TGF-β and other signaling mechanisms (Fig. 4H). Collectively, these findings suggest that DNA methylation levels in the hearts of recipients are altered, with hypomethylated DMGs potentially linked to DCM-HF.

### Identification of candidate DEGs regulated by DNA methylation in the promoter region

**Fig. 5:**
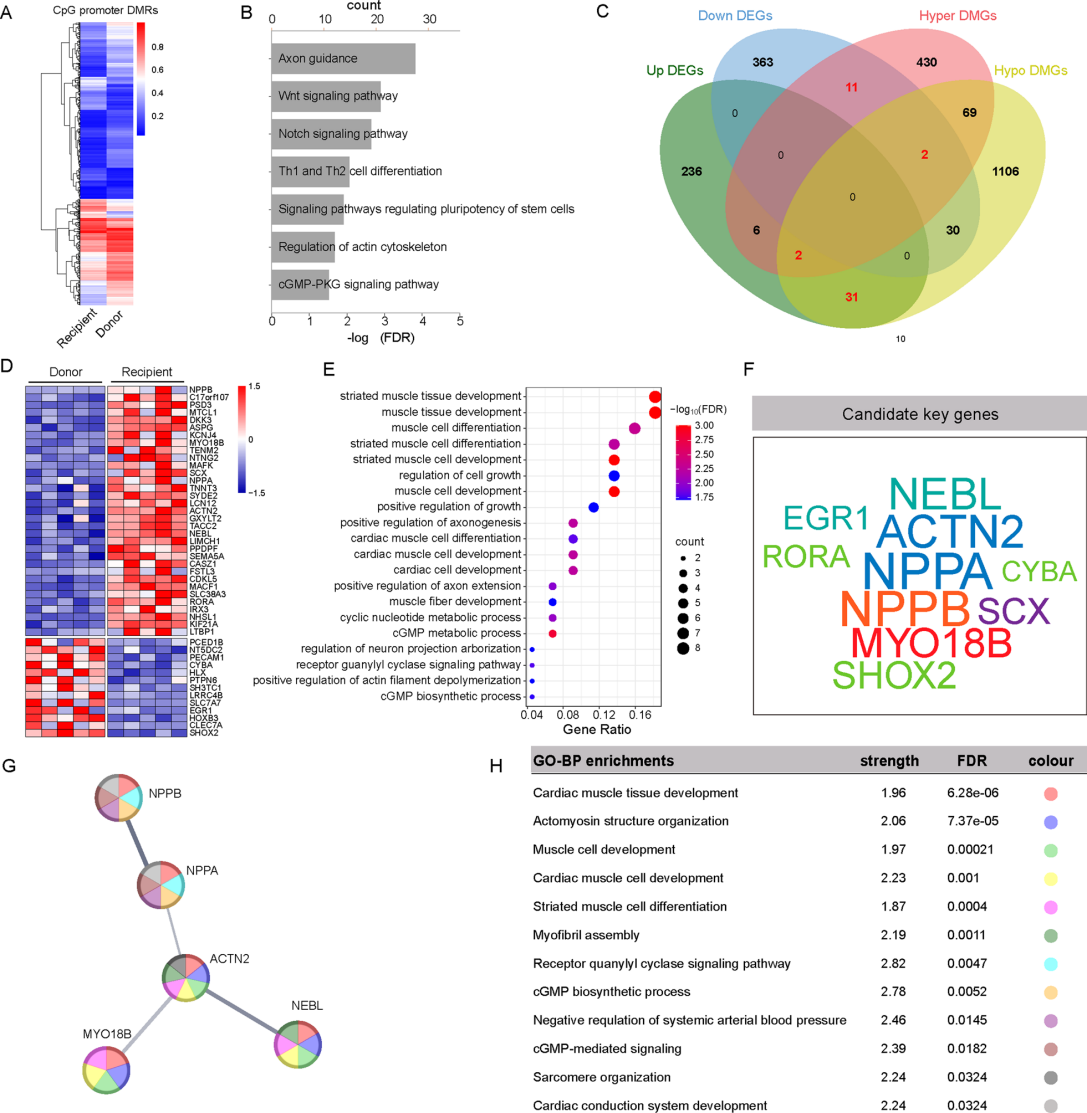
Correlation studies of DMRs-related Genes (DMGs) and Differentially Expressed Genes (DEGs). **A** Heatmaps showing DNA methylation levels of DMRs in promoter regions of Recipient and Donor group. **B** KEGG enrichment of DMGs in promoter regions. **C** Venn diagrams displaying the overlap between DEGs and DMGs in promoter regions. **D** Heatmap depicting the mRNA expression levels of 46 overlapped genes. **E** GO-BP enrichment of the overlapped genes. **(F)** 10 screened candidate key genes involved in enriched GO-BP terms. **G** and **H** Protein–protein Interaction (PPI) network (G, left) and the GO-BP enrichment (H, right) for the overlapped genes

Considering the negative regulatory effects of DNA methylation in the promoter region on gene expression, we conducted an integrative analysis of genes associated with DMRs in this region and DEGs. Among the identified DMRs, 3,185 were located in the promoter region (Table S3), influencing 1,614 genes involved in various pathways, including the Wnt signaling pathway, Notch signaling pathway, cGMP-PKG signaling pathway, regulation of the actin cytoskeleton, and axon guidance (Fig. 5A and B). We subsequently combined hypermethylated DMGs in the promoter region with downregulated DEGs, identifying 13 intersecting genes. Additionally, we combined hypomethylated DMGs in the promoter region with upregulated DEGs, resulting in the identification of 33 intersecting genes (Fig. 5C and D). By analyzing the frequency of occurrence of these genes, we ranked them and found that the top ten genes appeared most frequently and were most closely related to cardiac-related biological processes, including *NPPA*, *NPPB*, *ACTN2*, *NEBL*, *MYO18B*, *EGR1*, *RORA*, *CYBA*, *SHOX2*, and *SCX* (Fig. 5E and F). Through STRING protein–protein interaction and Gene Ontology biological process (GO-BP) analyses of 46 genes, we identified seven interacting genes: MYO18B, ACTN2, NPPA, NPPB, NEBL, SHOX2, and IRX3. Among these, MYO18B, ACTN2, NPPA, NPPB, and NEBL are closely associated with cardiac-related biological processes, such as striated muscle cell differentiation, myofibril assembly, and cGMP-mediated signaling. Additionally, transcriptome analysis indicated that the expression levels of SHOX2 and IRX3 were low (FPKM < 10), leading to their exclusion from further analysis. Notably, the retained five genes exhibit reduced DNA methylation in the promoter region, which correlates with increased gene expression (Fig. 5G and H). This observation implies that DEGs associated with DMRs in the promoter region may play a regulatory role in the pathological processes associated with DCM-HF.

### Validation of 5 key genes’ expression in the hearts

**Fig. 6:**
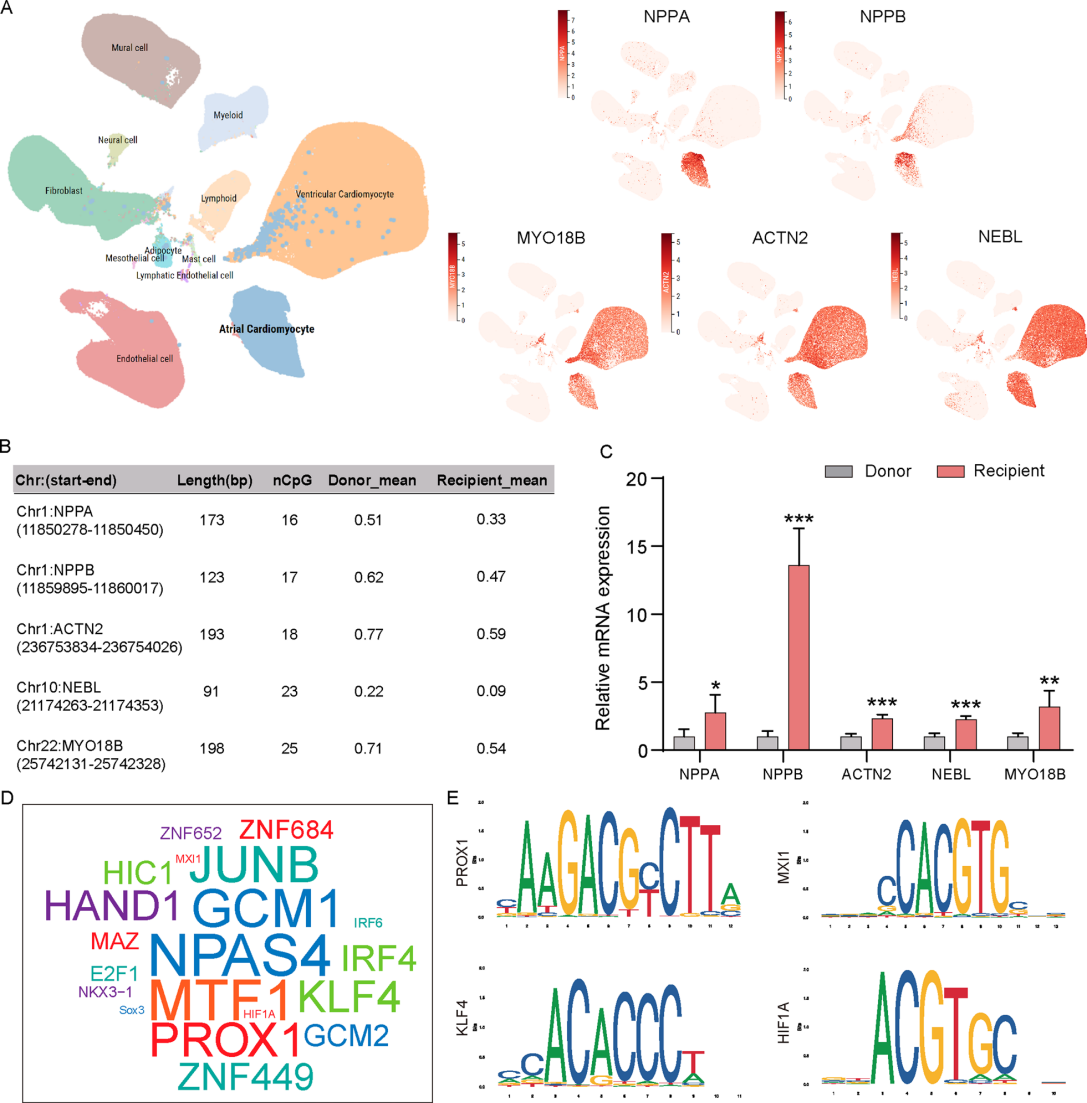
Validation of candidate genes associated with heart failure. **A** Feature plots of expression distribution for selected genes through Single cell RNA-sequencing (ScRNA-seq) analysis of the human heart cells. **B** Methylation levels in the promoter regions of the candidate genes. **C** RT-qPCR validation of the mRNA expression levels in Recipient and Donor groups. **D** Top20 identified transcription factors within the promoter regions of candidate genes using HOMER. **E** Candidate transcription factors associated with DCM-HF

To verify the expression of screened key genes in both the receptor and donor hearts, we initially utilized a human heart single cell online analysis tool (Heart Cell Atlas v2, https://www.heartcellatlas.org/)[22] to evaluate the expression of five specific genes across various heart subsets. Notably, these genes are predominantly expressed in cardiomyocytes, and the methylation levels of CpG sites in the promoter region of the recipient group are significantly lower than those in the donor group (Fig. 6A and B). Correspondingly, the mRNA expression levels in the recipient group are significantly higher than those in the donor group (Fig. 6C). To investigate transcription factors that may influence the methylation of the promoter region regulating gene expression pathways, we conducted transcription factor enrichment analyses of the promoter regions of these five genes using HOMER (v5.1.7), identifying the top 20 transcription factors (Fig. 6D). We subsequently refined this list to four candidate transcription factors based on their expression in the heart and the presence of binding motifs containing CpG sequences in the genomic DNA: PROX1, MXI1, KLF4, and HIF1A (Fig. 6E). These findings suggest that the reduction in DNA methylation levels in the promoter region may activate the expression of genes such as NPPA and NPPB by modulating the interactions of transcription factors like KLF4 and HIF1A, thereby contributing to the regulatory network associated with DCM-HF.

## Disscusion

Multi-omics analysis provides a comprehensive perspective on biological information, significantly enhancing our understanding of the complexity and dynamics of diseases from various angles. This approach elucidates the biomarker and molecular foundations of human diseases [23–26]. Typically, this analytical process involves genomic sequencing techniques, such as whole-genome sequencing, whole exome sequencing, and copy number variation sequencing, which clarify the origins of genetic diseases, including single nucleotide polymorphisms (SNPs), segmental deletions, and duplications. Additionally, epigenomic sequencing—which encompasses DNA methylation, RNA methylation, and chromatin accessibility—is employed to elucidate the pathogenesis of these diseases. Furthermore, expression profiling, including transcriptomics, proteomics, and metabolomics, is utilized to analyze downstream therapeutic targets associated with these conditions. This study integrates DNA methylation and RNA-sequencing to provide a comprehensive investigation of the epigenetic and transcriptomic changes related to dilated cardiomyopathy-related heart failure (DCM-HF). It offers essential insights into the relationship between DNA methylation in promoter regions and the resulting alterations in gene expression. By combining RNA-sequencing and whole-genome bisulfite sequencing analyses of atrial tissues from both DCM-HF patients and healthy donors, we identified 681 differentially expressed genes (DEGs) and 23,015 differentially methylated regions (DMRs). Among these, a subset of 46 hub genes exhibited coordinated changes in both methylation and expression. Notably, five genes—*NPPA*, *NPPB*, *ACTN2*, *NEBL*, and *MYO18B*—were identified as key contributors, demonstrating promoter hypomethylation alongside increased expression. These results underscore the significance of epigenetic dysregulation in cardiac stress-response pathways and structural remodeling, thereby providing new insights into the molecular mechanisms that drive the progression of DCM-HF.

### Changes in transcriptome and DNA methylation associated with DCM-HF

Among the 681 genes identified as significantly DEGs through transcriptome analysis, 406 were found to be upregulated and closely associated with pathways related to dilated cardiomyopathy. Conversely, 275 downregulated genes were linked to immune status, which plays a crucial regulatory role in heart failure (HF). Throughout the progression of heart failure, multiple immune cells within the heart-including macrophages, B cells, T cells, regulatory T cells, dendritic cells, natural killer cells, neutrophils, and mast cells-become activated. The activation of these immune cells may lead to an exacerbated inflammatory response, worsening myocardial injury and deteriorating cardiac function [27]. In contrast, specific immune markers, such as lymphocytes and the lymphocyte-to-monocyte ratio (LMR), are negatively correlated with the incidence of heart failure [28]. Moreover, lymphatic endothelial cells are activated following myocardial infarction, effectively creating an immunosuppressive microenvironment in the infarcted area by enhancing immunosuppressive functions, which suppress the proliferation of autoreactive CD8^+^ T cells and promote the expansion of reparative macrophages [29]. This immunosuppressive microenvironment helps mitigate further cardiac damage from autoimmune responses and facilitates cardiac repair. In our transcriptomic expression profiles, we did not observe an increase in the expression of classic pro-inflammatory factors such as *TNF*, *IL1B*, *IL6*, *CXCL8*, and *IL17*. This lack of increase can primarily be attributed to the FPKM values of these genes, which were all below 1, resulting in their exclusion from the analysis. Conversely, we noted a significant downregulation in the expression of genes associated with the proliferation and function of lymphocytes, including T cells, neutrophils, and monocytes, suggesting a potential presence of immune suppression. All patients in our study were in the late stages of chronic heart failure, leading us to speculate that the immune status in the hearts of heart failure patients may be influenced by the acute or chronic nature and duration of their condition. Furthermore, variations in omics technologies and donor characteristics may also impact the results of our analyses. The most significantly altered DEGs include transcription factors such as *HAND1* and *FOSB*, inflammatory regulatory proteins like *S100A9* and *SAA1*, as well as the muscle contraction protein *NEB*. These factors may play a crucial role in the pathological changes associated with DCM-HF. For instance, cardiomyocyte-specific overexpression of HAND1 can drive the pathogenesis of DCM through the genome-wide enhancer-promoter connectome, which is linked to decrease cardiomyocyte contractility and impaired calcium handling capacity, ultimately compromising cardiac function [30]. Moreover, the reduced expression of SAA1 may pose certain risks, including a weakened immune response and diminished cardiovascular protection [31]. Additionally, we compared the marker genes identified in the transcriptome with the plasma markers of heart failure documented in the literature [32, 33]. Our findings indicate that markers such as ANP, BNP, TNNT2, TNNI3, LTBP4, and FSTL3 align with existing reports. Notably, our examination of classic intracellular signaling pathways [34] revealed a significant activation of key genes in the PI3K/AKT pathway within the recipients. Although the fold changes in the expression of these genes were not pronounced, this finding suggests that intracellular signaling pathways, such as PI3K/AKT, may play a potential role in the pathogenesis of DCM-HF.

In studies of DNA methylation, DMRs-related genes (DMGs) are implicated in various biological processes and signaling pathways associated with DCM-HF. The DMGs with most significant alterations in promoter region include the transcription factors *PBX2* and *HOXA3*, the RNA-binding protein *RBFOX1*, the cell adhesion protein *PKP3*, the protein kinase *PHLDB1*, the GTPase-activating protein *AGAP2*, and the metabolism and immune regulatory protein *METRNL*. These alterations have been reported to potentially contribute to the progression of heart failure through mechanisms such as extracellular matrix remodeling, immune regulation, and transcriptional regulation. Furthermore, the identification of overlapping genes between hyper-DMGs and hypo-DMGs in the promoter region has revealed a total of 73 genes. The expression of these genes may be regulated by various transcription factors, and maintaining their expression homeostasis could be implicated in the pathological mechanisms underlying dilated cardiomyopathy (DCM). KEGG enrichment analysis indicates that these genes are associated with platelet activation and the relaxin signaling pathway, including *COL1A1*, *GNAS*, and *LCK*, among others. For instance, the overexpression of COL1A1 can lead to increased collagen deposition within myocardial tissue, resulting in myocardial stiffness and thickening, which adversely affects the heart’s normal function [35]. The GNAS protein plays a critical role in regulating cardiomyocyte contractility and heart rate, thereby influencing the heart’s pumping function and electrophysiological properties; its aberrant expression increases the risk of atherosclerosis and arrhythmia [36]. Additionally, LCK can interact with PKCε and undergo phosphorylation by it, enhancing the myocardium’s tolerance to ischemic injury [37].

### Integration of epigenetic and transcriptomic dysregulation

Our observation of widespread DNA hypomethylation in hearts affected by dilated cardiomyopathy-associated heart failure (DCM-HF) aligns with previous studies linking global methylation loss to various cardiovascular pathologies, including heart failure and myocardial infarction. The preferential hypomethylation of promoter regions in critical cardiac genes elucidates the mechanisms underlying the well-documented alterations associated with heart failure. Natriuretic peptides, particularly NPPA (translated to ANP) and NPPB (translated to BNP), have emerged as valuable predictors of stroke and heart failure, including NT-proBNP [38–40]. As a specific biomarker for heart failure, the elevated expression of NT-proBNP may correlate with reduced levels of DNA methylation in promoter regions. ACTN2 encodes an essential Z-disc protein critical for cross-linking actin filaments, and its dysregulation has been implicated in arrhythmias and contractile dysfunction [41]. The hypomethylation-driven overexpression of ACTN2 may initially stabilize sarcomeres but could eventually contribute to maladaptive hypertrophy. Similarly, NEBL, which anchors titin to actin, plays a pivotal role in diastolic function, and its altered expression may exacerbate ventricular stiffness [42]. MYO18B is crucial for maintaining the structural integrity of the myocardium in cardiomyocytes by regulating higher-order tissue organization and actin cross-linking within the sarcomere [43]. Dysregulation of MYO18B expression can lead to damage to the sarcomeric structure of the myocardium, subsequently impairing cardiac function and increasing the risk of cardiovascular disease. The prominence of these genes in protein–protein interaction networks further underscores their centrality in maintaining cardiomyocyte architecture, suggesting that their epigenetic dysregulation disrupts the delicate balance between compensatory remodeling and pathological decompensation.

The enrichment of transcription factors, including HIF1A and KLF4, in the promoter regions of hypomethylated genes indicates that hypoxia and metabolic stress are significant drivers of epigenetic reprogramming. HIF1A, a master regulator of hypoxic adaptation, is recognized for its ability to recruit DNA demethylases to specific genomic loci, which may explain the observed hypomethylation in stress-response genes [44]. Similarly, KLF4, which is essential in regulating cardiomyocyte differentiation and survival, may orchestrate methylation changes that influence the expression of target genes [45]. These interactions highlight the importance of investigating how environmental stressors, such as chronic ischemia and hemodynamic overload, dynamically affect the cardiac methylome in the context of dilated cardiomyopathy-associated heart failure (DCM-HF).

Smoking is recognized as one of the primary risk factors for cardiovascular diseases (CVD). Research indicates that smoking induces changes in DNA methylation and leads to abnormal expression of immune and inflammation-related genes, thereby increasing the prevalence of smoking-related diseases. Among these genes is the G-protein coupled receptor 15 (GPR15), which plays a critical role in regulating T-cell migration and immunity. Given that the male heart failure patients involved in our research exhibit varying degrees of smoking history, we assessed the expression and methylation levels of GPR15 alongside other reported smoking-related differentially expressed genes [46, 47]. Our findings indicate that these genes are largely not expressed in the heart. Future combined analysis of DNA methylation and transcriptome in the hearts of both smoking and non-smoking heart failure patients may provide more insights into the direct impact of smoking on DCM-HF.

### Limitations and future directions

While this study provides valuable insights, several limitations merit consideration. Firstly, the reliance on atrial tissues, rather than ventricular tissues where dilated cardiomyopathy-associated failure (DCM-HF) primarily occur, may limit the generalizability of the findings. Secondly, the small cohort size (n = 5 per group) restricts statistical power and highlights the necessity for validation in larger, multi-center cohorts. Thirdly, the correlative nature of our analysis prevents the establishment of causality between methylation changes and gene expression; therefore, functional studies utilizing CRISPR-based methylation editing or demethylating agents are crucial to confirm these regulatory relationships. Lastly, longitudinal analyses are needed to determine whether these methylation signatures serve as drivers of disease progression or are merely secondary consequences of heart failure.

### Translational perspectives

The reversible nature of DNA methylation positions it as an attractive therapeutic target. Hypomethylation of the promoters of *NPPA*, *NPPB*, *ACTN2*, *NEBL*, and *MYO18B* may serve as biomarkers for the early detection of heart failure (HF). Additionally, demethylation inhibitors and editing technologies that target maladaptive pathways have the potential to mitigate adverse remodeling [48]. Furthermore, the identification of KLF4 and HIF1A as upstream regulators opens avenues for therapies modulating transcription factor activity to restore epigenetic homeostasis.

## Conclusion

In summary, our integrative multi-omics approach elucidates the crucial role of promoter methylation in regulating cardiac stress-response and structural genes in dilated cardiomyopathy -associated heart failure (DCM-HF). By establishing a link between epigenetic dysregulation and subsequent transcriptional and functional alterations, this study deepens our understanding of the pathogenesis of heart failure and identifies novel targets for diagnostic and therapeutic innovation. Future research should prioritize the functional validation of these epigenetic drivers and their translation into clinical applications for managing DCM-HF.

## Supplementary Information

Additional file1 (XLSX 9 KB)

Additional file2 (XLSX 41 KB)

Additional file3 (XLSX 335 KB)

## Data Availability

The datasets used in the current study are available from the corresponding author on reasonable request.
